# Meta-analysis of the prevalence of Carpal Tunnel Syndrome (CTS) among dental health care personnel

**DOI:** 10.12688/f1000research.131659.1

**Published:** 2023-03-08

**Authors:** Deepika Chenna, Medhini Madi, Mathangi Kumar, Vijay Kumar, Sitaram Chopperla, Abhinav Tadikonda, Kalyana Pentapati

**Affiliations:** 1 Department of Immunohematology and Blood Transfusion, Kasturba Medical College, Manipal, Manipal Academy of Higher Education, Manipal, Karnataka, 576104, India; 2 Department of Oral Medicine and Radiology, Manipal College of Dental Sciences, Manipal, Manipal Academy of Higher Education, Manipal, Karnataka, 576104, India; 3 Public Health Dentistry, Amrita School of Dentistry, Amrita Vishwa Vidhyapeetham, Kochi, Kerala, India; 4 Department of Orthopedics, Kasturba Medical College, Manipal, Manipal Academy of Higher Education, Manipal, Karnataka, 576104, India; 5 Public Health Dentistry, Sri Sai College of Dental Surgery, Vikarabad, Telangana, India; 6 Department of Public Health Dentistry, Manipal College of Dental Sciences, Manipal, Manipal Academy of Higher Education, Manipal, Karnataka, 576104, India

**Keywords:** Carpal Tunnel, Pain, Dentist, Dentist, Dental students, Dental auxiliaries

## Abstract

**Background: **Carpal Tunnel Syndrome (CTS) is one such common disorder among dental health care personnel caused due to the entrapment neuropathy of the median nerve in the carpal tunnel. We aimed to evaluate the pooled estimates of the CTS among dental healthcare personnel.

**Methods:** We systematically reviewed the existing literature from six databases till January 1 ^st^, 2022. Studies reported in English along with the prevalence of CTS or where prevalence could be calculated were included. Independent screening of title and abstracts, and the full text was done by two examiners. Information collected was authors, year of publication, geographic location, type of dental healthcare personnel, sample size, distribution of age, sex, CTS, method of diagnosis, and risk of bias. The random effect model was used to estimate the pooled estimates.

**Results:** Thirty-seven studies yielded 38 estimates. A total of 17,152 dental health care personnel were included of which 2717 had CTS. The overall pooled prevalence of CTS among the included studies was 15%, with a high heterogeneity. Meta-analysis showed no significant difference in the pooled estimates of CTS between male and female dental healthcare personnel (OR: 0.73; 95% CI: 0.52 -1.02; I ^2^= 69.71). The pooled estimates among the dentist and dental auxiliaries were 20% and 10%, respectively. The pooled prevalence of CTS with self-reported measures, clinical examination and NCS were 21%, 13% and 8% respectively. Meta-regression showed that the prevalence estimates were significantly associated with publication year (coefficient: 0.006; 95% CI= 0.002-0.01).

**Conclusion:** One out of seven dental health care personnel may be affected by CTS. No significant difference was seen in the prevalence of CTS between male and female dental healthcare personnel.

## Introduction

Dentistry involves complex procedures with repetitive movements, firm grasp, and fine tactile movements with prolonged static postures often with poor illumination and access. Due to this dental healthcare personnel are prone to various musculoskeletal disorders. ^1^^–^^7^

Carpal Tunnel Syndrome (CTS) is one such common disorder among dental health care personnel caused due to the entrapment neuropathy of the median nerve in the carpal tunnel. It can cause sensorimotor symptoms like pain, numbness, tingling, and weakness in the hand leading to loss of grip strength and dexterity. CTS can have negative effects on the individual quality of life, functional disability, limitation of daily living, poor sleep quality, decreased productivity, and the discontinuation of the profession. It can have a significant impact on the individual’s family and the community.

Numerous risk factors like repetitive actions, ^8^ use of vibrating instruments, ^8^^,^^9^ pregnancy, diabetes, ^10^ obesity, ^10^ trauma, smoking, ^11^ increasing age, ^8^^,^^12^ female sex, ^9^^,^^10^^,^^13^^–^^16^ wrist diameter ratio, ^9^ clinical experience ^12^ and the number of working hours per day ^14^ have been linked to the development of CTS. Studies have used different modalities for the assessment of CTS. Self-reported measures (for ex: Boston carpal tunnel questionnaire, Nordic questionnaire, hand diagram, Clinical questionnaire by Kamath and Stothard) are the most used methods of assessment. This was followed by nerve conduction studies (NCS) and clinical examination using variety of tests (Tinel’s test, Phalen’s test, or Durkan compression test) and a combination of any of the above methods.

The prevalence of CTS among dental healthcare personnel was reported to be high akin to musculoskeletal disorders when compared to the general population. However, no attempt was made to consolidate the estimates of CTS among dental healthcare personnel. Considering this, our goal was to compile the estimates of the CTS among dental healthcare professionals reported from the literature.

## Methods

We systematically reviewed the existing literature to evaluate the prevalence of CTS among dental healthcare personnel. The protocol for this study was registered with “International Platform of Registered Systematic Review and Meta-analysis Protocols” (INPLASY202210084) ^17^ and was reported as per the “PRISMA” guidelines.

### Search strategy

A methodical search of six databases (“PubMed, Embase, Dentistry and Oral Sciences Source, CINAHL, Web of Science, and Scopus”) was conducted without any date restrictions till January 1 ^st^, 2022. The keywords used were “dentist” OR “dental student” OR “dental auxiliary” OR “dental hygienist” OR “dental personnel” AND “carpal tunnel syndrome” or “carpal tunnel” or “medial nerve entrapment” or “CTS.”

### Inclusion and exclusion criteria

Studies written in English that reported the prevalence of CTS or where the prevalence could be determined were included. Studies reported as letters, commentaries, and short communications were excluded.

### Screening

Studies obtained from various databases were added to “Rayyan – a web-based application” for duplicate removal and title and abstract screening. This was followed by full-text screening and data extraction. Two review authors did the screening independently, and the disagreements, if any, were resolved by a third review author.

### Data extraction

Two review authors independently performed the data extraction. Information that was collected was authors, year of publication, country, type of dental personnel, age and sex distribution, sample size, number of participants with CTS, method of diagnosis used (self-reported, clinical examination, or NCS), the sex distribution of CTS and risk of bias.

### Risk of bias assessments

Two review authors independently evaluated the risk of bias using a nine-item questionnaire developed by Hoy *et al.* ^18^ The total score was obtained based on which the studies were graded as low (0-3), moderate (4-6), or high risk of bias (7-9).

### Statistical analysis

All the analysis was done using OpenMeta software (Metafor Package 1.4, 1999). The random effect model (Restricted maximum likelihood method) was used to estimate the pooled estimates. Subgroup analysis was performed for the type of dental personnel, geographic location, and type of diagnosis. The distribution of the prevalence of CTS between males and females was evaluated using the Binary Random effect model, and the Odds ratio was calculated. Publication bias was assessed using a funnel plot and Fail-Safe N analysis using the Rosenthal approach. Meta-regression was done with publication year to evaluate time trends in the prevalence estimates. Sensitivity analysis was performed using the Leave one out method. Heterogeneity among the studies was assessed using I ^2^ statistics. Underlying data for this review is available at Mendeley datasets. ^19^

## Results

**Figure 1.**
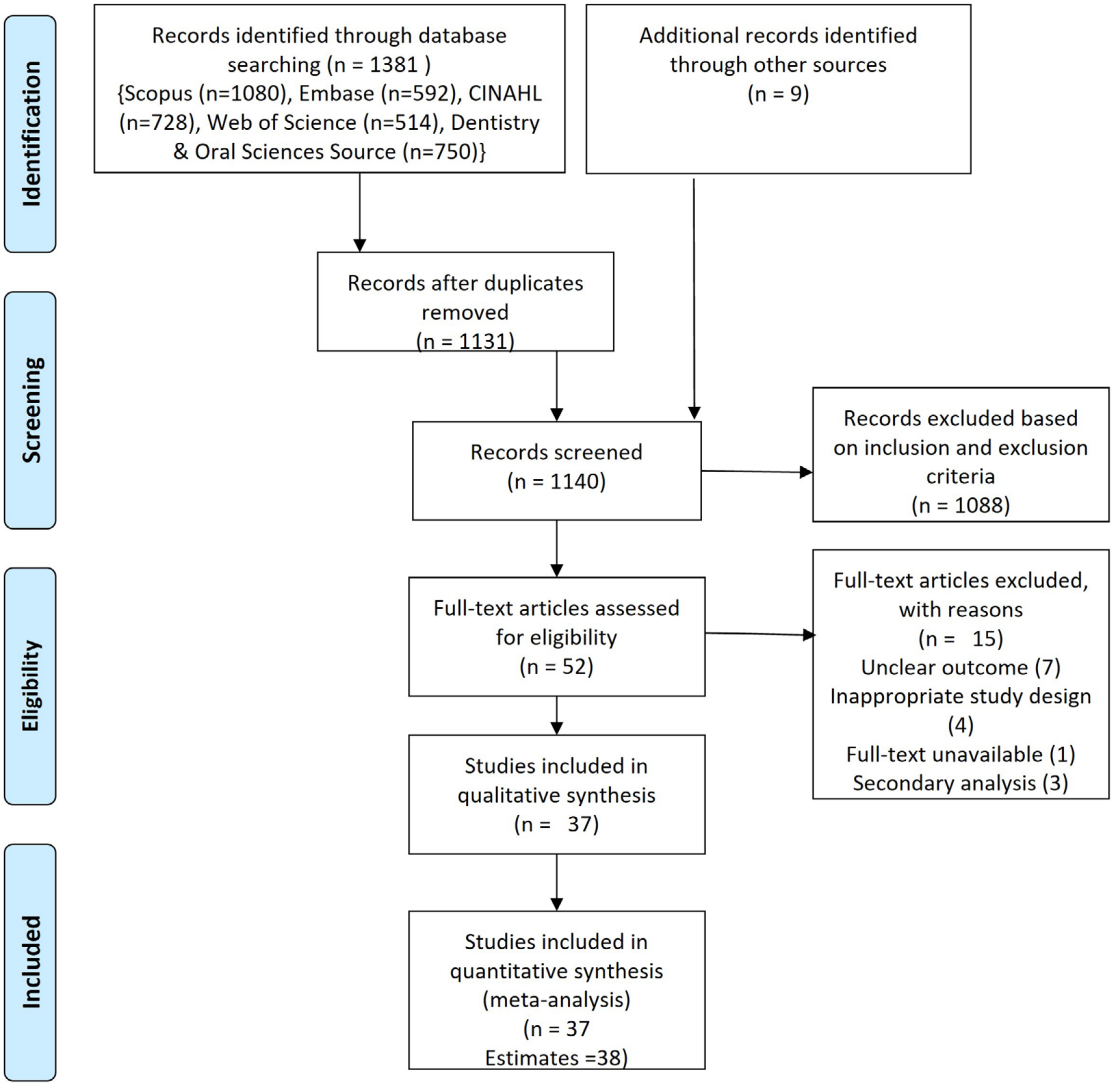
PRISMA flowchart.

The search of six databases (Embase (n=77), Scopus (n=54), PubMed (n=120), CINAHL (n=465), DOSS (n=570), and Web of Science (n=95)) yielded 1381 studies, of which 249 were duplicates. A total of 1131 studies were subjected to title, and abstract screening out of which 43 studies were eligible for full-text screening. Another nine studies were obtained from manual searching of reference lists at the end of publications resulting in a total of 52 studies for full-text screening. After screening full-text, 15 studies were further excluded due to missing outcome (n=7), the secondary publication (n=3), or inappropriate study design (n=4) and full-text unavailable (n=1). ^20^ Data extraction was performed for 37 studies which yielded 38 estimates ( Figure 1, Table 1). ^9^^,^^12^^–^^16^^,^^21^^–^^51^

**Table 1.** Characteristics of the included studies.

Author, Year	Continent	Diagnosis	Risk of bias	N	Prevalence (%)	Type of dental health care personnel
Macdonald *et al.* 1988	NA	SR	L	2464	8.69	DA
Osborn *et al.* 1990	NA	SR	M	394	6.85	DA
Conrad *et al.* 1991	NA	V	L	20	0.00	DA
Conrad *et al.* 1992	NA	V	M	16	0.00	DA
Conrad *et al.* 1993	NA	V	L	16	0.00	DA
Nakladalova *et al.* 1995	Eu	NC	M	120	3.33	DA
Liss *et al.* 1995	NA	SR	M	1058	10.21	DA
Scoggins and Campbell 1995	NA	SR	M	79	5.06	DA
Rice *et al.* 1996	NA	PE	L	45	11.11	Mixed
Akesson *et al.* 1999	Eu	PE	L	84	7.14	Mixed
Lalumandier *et al.* 2000	NA	SR	L	5115	25.45	Mixed
Hamann *et al.* 2001	NA	NC	L	1079	4.82	D
Anton *et al.* 2002	NA	NC	L	89	8.99	DA
Werner *et al.* 2002	NA	NC	L	305	5.57	DA
Mamatha *et al.* 2005	Asia	SR	L	300	32.00	D
Werner *et al.* 2005	NA	NC	L	232	0.43	D
Werner *et al.* 2005b	NA	SR	L	111	0.90	DA
Cherniack *et al.* 2006	NA	PE	L	160	12.50	DA
Greathouse *et al.* 2009	NA	NC	L	35	25.71	DA
Shaffer *et al.* 2012	NA	NC	L	55	10.91	DA
Haghighat *et al.* 2012	Asia	PE	L	240	16.67	D
Borhan *et al.* 2013	Asia	NC	L	40	17.50	D
Khan *et al.* 2014	Asia	SR	L	417	10.31	D
Pai *et al.* 2014	Asia	SR	L	210	20.00	D
Munirah *et al.* 2014	Asia	SR	M	99	21.21	D
Hodacova *et al.* 2015	Eu	SR	M	575	14.61	D
Nor Rasid *et al.* 2016	Asia	SR	L	95	38.95	DA
Ehsan *et al.* 2016	Asia	PE	L	103	15.53	D
Prasad *et al.* 2017	Asia	SR	L	100	86.00	D
Jaoude *et al.* 2017	Asia	SR	L	314	7.64	D
De JeSUS *et al.* 2018	SA	SR	L	286	13.29	D
Inbasekharan *et al.* 2018	Asia	SR	L	120	25.83	D
Alhusain *et al.* 2019	Asia	SR	L	223	30.49	D
Meisha *et al.* 2019	Asia	SR	L	234	9.40	D
Al Muraikhi *et al.* 2020	Asia	SR	L	66	24.24	D
Harris *et al.* 2020	NA	SR	L	647	18.39	DA
Berdouses *et al.* 2020	Eu	SR	L	1500	8.27	D
Maghsoudipour *et al.* 2021	Asia	NC	L	106	17.92	D

NA: North America; SA: South America; Eu: Europe; SR: self-reported; NC: Nerve conduction; PE: Physical examination; V: Vibrometry; DA: Dental Auxilaries; D: Dentists/dental students; N: sample size; Mixed: Dental Auxiliaries or Dentists/dental students.

### Prevalence

**Figure 2.**
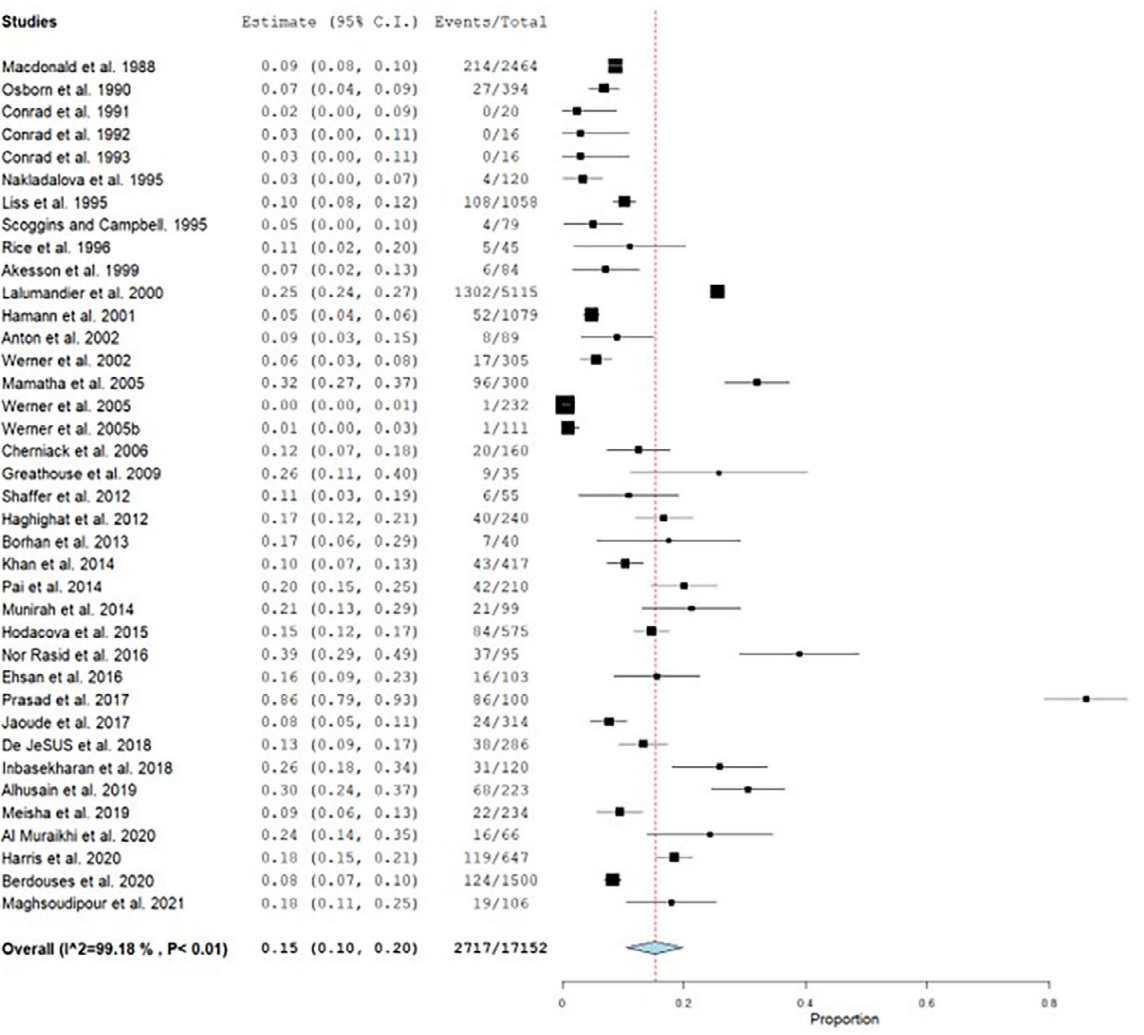
Forest plot showing the pooled prevalence of CTS.

A total of 17,152 dental health care personnel were included in 37 studies of which 2717 had CTS. The prevalence ranged from 0 to 86%. ^21^^,^^42^^,^^50^^,^^51^ The overall pooled prevalence of CTS was 15%, with a high heterogeneity (I ^2^=99.18) ( Figure 2).

### Age

Nine studies have not reported the age distribution. ^14^^,^^16^^,^^25^^,^^30^^,^^44^^–^^46^^,^^48^^,^^49^ The age-specific estimates of CTS lacked uniformity in reporting. The mean age ranged from 21-50 years.

### Sex

**Figure 3.**
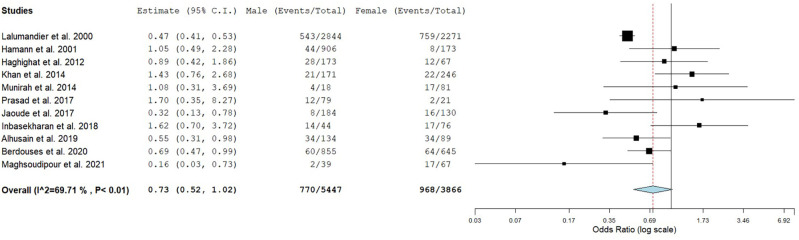
Forest plot showing the sex differences in the prevalence of CTS.

Eight studies have not reported the sex distribution of the participants. ^21^^,^^22^^,^^26^^,^^30^^,^^48^^–^^51^ Twelve studies reported the prevalence of CTS concerning the sex of which one study had only female participants and was excluded from analysis. ^12^ Meta-analysis showed no significant difference in the pooled estimates of CTS between male and female dental healthcare personnel (OR: 0.73; 95% CI: 0.52-1.02; P=0.07; I ^2^=69.71) ( Figure 3).

### Geographic location

Almost half of the studies were reported from North America (n=17) ^12^^,^^16^^,^^21^^–^^23^^,^^25^^,^^27^^–^^29^^,^^31^^,^^32^^,^^34^^,^^46^^,^^48^^–^^51^ followed by Asia (n=15) ^9^^,^^14^^,^^15^^,^^30^^,^^33^^,^^35^^–^^39^^,^^41^^–^^43^^,^^45^^,^^47^ and Europe (n=4). ^13^^,^^24^^,^^26^^,^^40^ Only one study was reported from South America. ^44^ High pooled prevalence was seen among studies that were reported from Asia (25%), followed by North America (9%) and Europe (8%) ( Table 2).

**Table 2.** Subgroup analysis of the pooled estimates of overall MSD.

Characteristic	Estimate (95% CI)	Q	I ^2^	Number of estimates
Overall	0.15 (0.10-0.2)	2073.13	99.18	38
Sex				
Male	0.14 (0.09-0.19)	265.61	96.41	11
Female	0.17 (0.11-0.23)	417.15	95.44	12
Dental personnel				
Dentists	0.2 (0.12-0.28)	1045.42	99.4	19
Dental auxiliaries	0.1 (0.05-0.14)	182.68	96.6	16
Mixed	0.15 (0.03-0.27)	48.76	93.92	3
Continent				
North America	0.09 (0.05-0.12)	1291.72	97.61	18
Europe	0.08 (0.04-0.13)	27.46	90.64	4
Asia	0.25 (0.15-0.35)	546.87	97.96	15
Risk of bias				
Low	0.17 (0.11-0.22)	2028.49	99.33	31
Moderate	0.09 (0.05-0.13)	44.53	91.23	7
Method of diagnosis				
Self-reported	0.21 (0.13-0.29)	1154.82	99.4	20
Physical examination	0.13 (0.09-0.16)	7.36	47.12	5
Nerve conduction studies	0.08 (0.03-0.12)	85.93	95.49	10

### Type of dental personnel

More than half of the included studies were reported among dentists (n=18) ^9^^,^^13^^–^^15^^,^^27^^,^^28^^,^^30^^,^^33^^,^^35^^–^^40^^,^^42^^–^^44^^,^^47^ followed by dental auxiliaries (n=16). ^12^^,^^21^^–^^24^^,^^27^^,^^28^^,^^31^^,^^32^^,^^34^^,^^41^^,^^48^^–^^51^ The pooled estimates among the dentist and dental auxiliaries were 20% and 10%, respectively ( Table 2).

### Method of diagnosis

The majority of the included studies (n=21) had used only self-reported measures for estimating the prevalence of CTS. ^13^^–^^16^^,^^22^^,^^23^^,^^29^^,^^30^^,^^37^^–^^49^ Nine studies have used nerve conduction studies ^9^^,^^12^^,^^24^^,^^27^^–^^29^^,^^32^^,^^34^^,^^36^ out of which four studies used clinical examination along with NCS. ^9^^,^^32^^–^^34^ Only five studies have used clinical examination. ^25^^,^^26^^,^^31^^,^^33^^,^^35^ Three studies conducted have used Vibrometry and have reported nil prevalence. ^21^^,^^50^^,^^51^ The pooled prevalence of CTS with self-reported measures, clinical examination and NCS were 21%, 13% and 8% respectively ( Table 2).

### Risk of bias

Majority of the studies (n=30) were in the low-risk category with a pooled prevalence of 17% ( Tables 1 and 2). ^9^^,^^12^^–^^16^^,^^25^^–^^36^^,^^38^^,^^39^^,^^41^^–^^48^^,^^50^^,^^51^

### Publication bias

**Figure 4.**
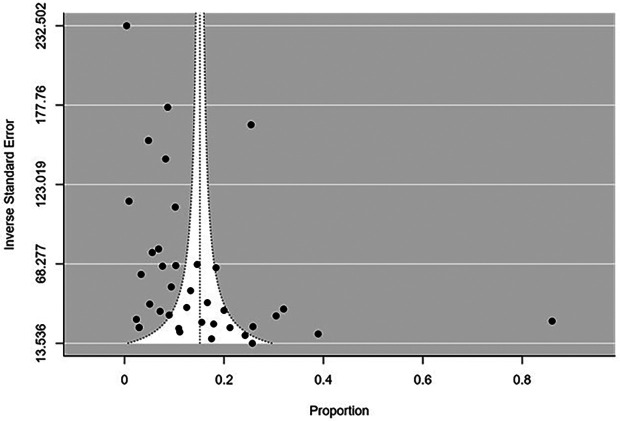
Funnel plot for publication bias.

The funnel plot showed publication bias (Fail safe N=26129; P-value<0.001) ( Figure 4).

### Sensitivity analysis

There was no change in the overall pooled estimate of CTS using the Leave-one out method.

### Meta-regression

**Figure 5.**
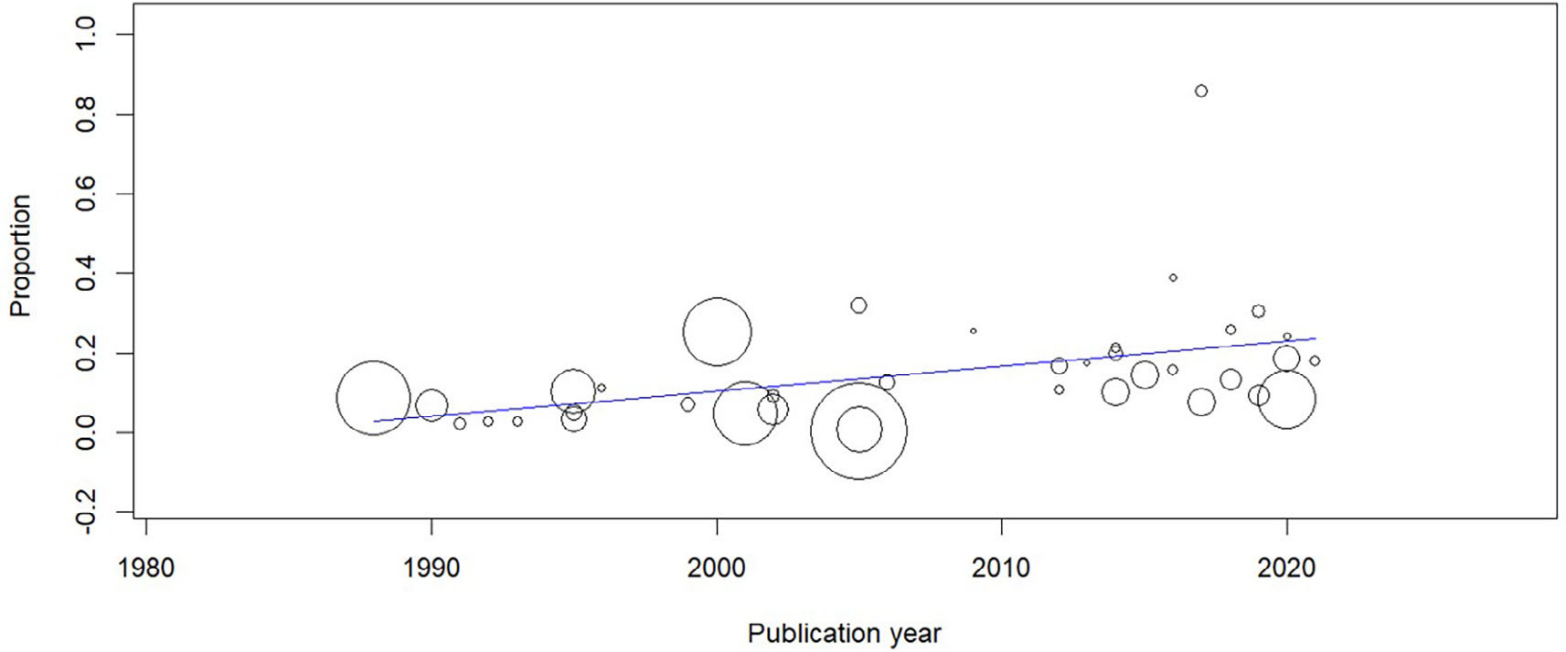
Meta-regression showing the time trends in the prevalence of CTS.

A meta-regression was performed to evaluate the pooled estimates of CTS with publication year. The prevalence estimates were significantly associated with publication year (coefficient: 0.006; 95% CI=0.002-0.01; P=0.002) ( Figure 5).

## Discussion

We conducted a systematic review of the prevalence of CTS among dental healthcare personnel. Many systematic reviews reported a high prevalence of musculoskeletal disorders among these professionals ^1^^–^^7^ without emphasizing the CTS.

High heterogeneity was observed among the studies that were included in this review. The overall pooled prevalence of CTS was 15% obtained from 38 estimates. It was higher among dentists than dental auxiliaries. The prevalence was higher than the reported studies among other professionals (9.6%). ^52^^,^^53^ The age-standardised prevalence rates of confirmed clinical and NCS were 2.1 and 3% among males and females, respectively. ^54^ A study among Danish office workers reported a confirmed CTS prevalence of 5%. ^55^ It was reported that repetitive activity and firm gripping could be a major risk factor for the development of CTS. ^52^ This suggests that dental healthcare personnel have a higher risk of CTS than the general population. In our analysis, only six studies reported a prevalence of less than 5%. ^21^^,^^24^^,^^27^^,^^29^^,^^50^^,^^51^ More than half of the studies showed higher than 10% prevalence. ^9^^,^^14^^,^^16^^,^^23^^,^^25^^,^^30^^–^^44^^,^^46^^,^^47^ There was substantial variation in the estimates of CTS with geographic location. Studies reported from Asia showed a high pooled prevalence of CTS.

The pooled prevalence among male and female dental healthcare personnel was 14 and 17%, respectively. Few studies have reported female predilection to CTS among dental healthcare personnel ^9^^,^^13^^–^^16^ and the general population. ^56^ However, we found no significant difference between male and female dental healthcare personnel.

There were substantial variations in the assessment of CTS among the included studies. Methods like self-reported measures, clinical examination (Tinels test, Phalen’s test, or compression test), Vibrometry and NCS were used for the assessment of CTS. Studies that used self-reported measures showed higher pooled prevalence than those studies that used clinical examination and NCS for the diagnosis of CTS. NCS is a useful tool and can be used as complimentary methods with clinical examination in the assessment of CTS. It is not recommended to be used as a sole method of diagnosis as it has limitations like difficulty in the assessment of nerve injuries that are very distal or proximal to the extremity, timing of the test, expertise of the examiner, multi-level injury along the course of nerve or systemic polyneuropathy. Also, the nerve latency is mainly due to the available myelinated fibers than the affected fibers. Due to the above reasons, a thorough physical examination of hand is a prerequisite for the diagnosis of CTS.

Our review included studies over four decades and it was seen that there was an increasing trend in the prevalence estimates of CTS. This could be attributed to many factors like increasing workload, increasing awareness about CTS, comorbidities etc.

Further high-quality studies using clinical examination for the identification of CTS among a representative sample of dental health care personnel using STROBE guidelines are required for calculating robust prevalence estimates. High heterogeneity among the included studies, inclusion of only studies that were reported in English, lack of age specific estimates, variations in the assessment of CTS are some of the limitations.

## Conclusion

One out of seven dental health care personnel may be affected by CTS. There was no difference in the prevalence of CTS between male and female dental healthcare personnel. Dentists more than dental auxiliaries are affected by CTS.

## Data Availability

Mendeley Data: Pooled prevalence of Carpal Tunnel syndrome among dental health care providers, https://doi.org/10.17632/m2tytmjdzf.2. ^19^ This project contains the following underlying data:
- Data CTS mendeley.xlsx Mendeley Data: PRISMA checklist ‘ *Pooled prevalence of Carpal Tunnel syndrome among dental health care providers*’, https://doi.org/10.17632/m2tytmjdzf.2. ^19^ Data are available under the terms of the Creative Commons Attribution 4.0 International license (CC-BY 4.0).

## References

[1] ZakerJafari HR YektaKooshali MH : Work-Related Musculoskeletal Disorders in Iranian Dentists: A Systematic Review and Meta-analysis. *Saf. Health Work.* 2018;9(1):1–9. 10.1016/j.shaw.2017.06.006 30363086 PMC6111132

[2] Leggat PA Kedjarune U Smith DR : Occupational health problems in modern dentistry: A review. *Ind. Health.* 2007;45(5):611–621. 10.2486/indhealth.45.611 18057804

[3] Puriene A Janulyte V Musteikyte M : General health of dentists. Literature review. *Stomatologija.* 2007;9(1):10–20. 17449973

[4] Hayes MJ Cockrell D Smith DR : A systematic review of musculoskeletal disorders among dental professionals. *Int. J. Dent. Hyg.* 2009;7(3):159–165. 10.1111/j.1601-5037.2009.00395.x 19659711

[5] Shams-Hosseini NS Vahdati T Mohammadzadeh Z : Prevalence of Musculoskeletal Disorders among Dentists in Iran: A Systematic Review. *Mater. Sociomed.* 2017;29(4):257–262. 10.5455/MSM.2017.29.257-262 29284995 PMC5723169

[6] Chenna D Pentapati KC Kumar M : Prevalence of musculoskeletal disorders among dental healthcare providers: A systematic review and meta-analysis. *F1000Res.* 2022;11:1062. 10.12688/f1000research.124904.1 36505095 PMC9709350

[7] Lietz J Kozak A Nienhaus A : Prevalence and occupational risk factors of musculoskeletal diseases and pain among dental professionals in Western countries: A systematic literature review and meta-analysis. *PLoS One.* 2018;13(12):e0208628. 10.1371/journal.pone.0208628 30562387 PMC6298693

[8] Spahn G Wollny J Hartmann B : Metaanalysis for the evaluation of risk factors for carpal tunnel syndrome (CTS) Part II. Occupational risk factors. *Z. Orthop. Unfall.* 2012;150(5):516–524. 10.1055/S-0032-1315346 23076750

[9] Maghsoudipour M Hosseini F Coh P : Evaluation of occupational and non-occupational risk factors associated with carpal tunnel syndrome in dentists. *Work.* 2021;69(1):181–186. 10.3233/WOR-213467 33998581

[10] Becker J Nora DB Gomes I : An evaluation of gender, obesity, age and diabetes mellitus as risk factors for carpal tunnel syndrome. *Clin. Neurophysiol.* 2002;113(9):1429–1434. 10.1016/S1388-2457(02)00201-8 12169324

[11] Pourmemari MH Viikari-Juntura E Shiri R : Smoking and carpal tunnel syndrome: a meta-analysis. *Muscle Nerve.* 2014;49(3):345–350. 10.1002/MUS.23922 23761223

[12] Anton D Rosecrance J Merlino L : Prevalence of musculoskeletal symptoms and carpal tunnel syndrome among dental hygienists. *Am. J. Ind. Med.* 2002;42(3):248–257. 10.1002/ajim.10110 12210693

[13] Berdouses E Katsantoni A Andrikoula T : Work-Related Musculoskeletal Disorders among Greek Dentists - A Nationwide Survey. *Dent. Res. Oral Heal.* 2020;03(04):169–181. 10.26502/droh.0031

[14] Alhusain FA Almohrij M Althukeir F : Prevalence of carpal tunnel syndrome symptoms among dentists working in Riyadh. *Ann. Saudi Med.* 2019;39(2):104–111. 10.5144/0256-4947.2019.07.03.1405 30905925 PMC6464669

[15] Bou Jaoude S Naaman N Nehme E : Work-Related musculoskeletal pain among lebanese dentists: An epidemiological study. *Niger. J. Clin. Pract.* 2017;20(8):1002–1009. 10.4103/NJCP.NJCP_401_16 28891546

[16] Lalumandier JA McPhee SD Riddle S : Carpal tunnel syndrome: Effect on Army dental personnel. *Mil. Med.* 2000;165(5):372–378. 10.1093/milmed/165.5.372 10826385

[17] Pentapati K Chenna D Kumar M : Prevalence of Carpal Tunnel Syndrome among Dental Health Care Providers-Systematic Review Protocol. 2022. 10.37766/inplasy2022.1.0084 PMC10372462 37521768

[18] Hoy D Brooks P Woolf A : Assessing risk of bias in prevalence studies: modification of an existing tool and evidence of interrater agreement. *J. Clin. Epidemiol.* 2012;65(9):934–939. 10.1016/j.jclinepi.2011.11.014 22742910

[19] Pentapati K Chenna D : Pooled prevalence of Carpal Tunnel syndrome among dental health care providers.[Dataset]. *Mendeley Data.* 2023;V2. 10.17632/m2tytmjdzf.2 PMC10372462 37521768

[20] Ravisankar A Thenmozhi MS : Awareness, knowledge, and prevalence of carpal tunnel syndrome among dental students in Saveetha Dental College. *Drug Invent. Today.* 2020;14(3):151–154.

[21] Conrad JC Conrad KJ Osborn JB : A short-term, three-year epidemiological study of median nerve sensitivity in practicing dental hygienists. *J. Dent. Hyg.* 1993;67(5):268–272. 8270995

[22] Scoggins KM Campbell RM : Impact of carpal tunnel education on changing dental hygienists knowledge, risk behaviors, symptoms and functional performance. *Work.* 1995;5(4):243–254. 10.3233/WOR-1995-5402 24441376

[23] Liss GM Jesin E Kusiak RA : Musculoskeletal problems among Ontario dental hygienists. *Am. J. Ind. Med.* 1995;28(4):521–540. 10.1002/AJIM.4700280408 8533793

[24] Nakladalova M Fialova J Korycanova H : State of health in dental technicians with regard to vibration exposure and overload of upper extremities. *Cent. Eur. J. Public Health.* 1995;3 Suppl:129–131. 9150992

[25] Rice VJ Nindl B Pentikis JS : Dental workers, musculoskeletal cumulativetrauma, and carpal tunnel syndrome: Who is at risk? a pilot study. *Int. J. Occup. Saf. Ergon.* 1996;2(3):218–233. 10.1080/10803548.1996.11076350 10602587

[26] Åkesson I Johnsson B Rylander L : Musculoskeletal disorders among female dental personnel--clinical examination and a 5-year follow-up study of symptoms. *Int. Arch. Occup. Environ. Health.* 1999;72(6):395–403. 10.1007/S004200050391 10473839

[27] Hamann C Werner RA Franzblau A : Prevalence of carpal tunnel syndrome and median mononeuropathy among dentists. *J. Am. Dent. Assoc.* 2001;132(2):163–170. 10.14219/JADA.ARCHIVE.2001.0150 11217588

[28] Werner RA Hamann C Franzblau A : Prevalence of carpal tunnel syndrome and upper extremity tendinitis among dental hygienists. *J. Dent. Hyg.* 2002;76(2):126–132. 12078576

[29] Werner RA Franzblau A Gell N : Prevalence of upper extremity symptoms and disorders among dental and dental hygiene students. *J. Calif. Dent. Assoc.* 2005;33(2):123–131. 15816702

[30] Mamatha Y Gopikrishna V Kandaswamy D : Carpal tunnel syndrome: survey of an occupational hazard. *Indian J. Dent. Res.* 2005;16(3):109–113. 16454325

[31] Cherniack M Brammer AJ Nilsson T : Nerve conduction and sensorineural function in dental hygienists using high frequency ultrasound handpieces. *Am. J. Ind. Med.* 2006;49(5):313–326. 10.1002/AJIM.20288 16570257

[32] Greathouse DG Root TM Carrillo CR : Clinical and electrodiagnostic abnormalities of the median nerve in dental assistants. *J. Orthop. Sports Phys. Ther.* 2009;39(9):693–701. 10.2519/jospt.2009.2995 19721216

[33] Haghighat A Khosrawi S Kelishadi A : Prevalence of clinical findings of carpal tunnel syndrome in Isfahanian dentists. *Adv. Biomed. Res.* 2012;1(1):13. 10.4103/2277-9175.96069 23210072 PMC3507010

[34] Shaffer SW Moore R Foo S : Clinical and electrodiagnostic abnormalities of the median nerve in US Army Dental Assistants at the onset of training. *U.S. Army Med. Dep. J.* 2012;72–81. 22815168

[35] Ehsan MA Ehsan S Arshad HS : Frequency of Carpal Tunnel Syndrome in Dentists Working in Government Hospitals of Lahore. *Int. J. Sci. Res.* 2016;5(5):1672–1675.

[36] Borhan Haghighi A Khosropanah H Vahidnia F : Association of dental practice as a risk factor in the development of carpal tunnel syndrome. *J. Dent. (Shiraz, Iran).* 2013;14(1):37–40. PMC3927669 24724115

[37] Munirah M Normastura A Azizah Y : Prevalence of Probable Carpal Tunnel Syndrome and itsAssociated Factors among Dentists in Kelantan. *Int. J. Collab. Res. Intern. Med. Public Heal.* 2014;6(8):247–259.

[38] Pai MB Shenoy R Rao A : Symptoms of Carpal Tunnel Syndrome in a dental work force of a developing country. *Int. J. Adv. Res.* 2014;2(2):87–94.

[39] Ahmed KA Siddiqui AZ Ahmed MR : Prevalence of carpel tunnel syndrome in the dentists working in Karachi. *Pakistan Oral Dent J.* 2014;34(4):588–591.

[40] Hodacova L Sustova Z Cermakova E : Self-reported risk factors related to the most frequent musculoskeletal complaints among Czech dentists. *Ind. Health.* 2015;53(1):48–55. 10.2486/INDHEALTH.2013-0141 25327296 PMC4331194

[41] Nurfarah Wahidah MN Nurul SN Munirah MANAR : Probable carpal tunnel syndrome and its coping Hospital Universiti Sains Malaysia. *J. Sch. Dent. Sci. USM.* 2016;11(2):31–38.

[42] Prasad DA Appachu D Kamath V : Prevalence of low back pain and carpal tunnel syndrome among dental practitioners in Dakshina Kannada and Coorg District. *Indian J. Dent. Res.* 2017;28(2):126–132. 10.4103/IJDR.IJDR_672_16 28611320

[43] Inbasekaran D Sankari M Nambi G : Prevalence of carpal tunnel syndrome among dentists in Chennai, India. *Drug Invent. Today.* 2018;10(3):3262–3265.

[44] De JeSUS LC Tedesco TK MaceDo MC : A self-report joint damage and musculoskeletal disorders data among dentists: a cross-sectional study. *Minerva Stomatol.* 2018;67(2):62–67. 10.23736/S0026-4970.17.04033-X 29446269

[45] Meisha DE Alsharqawi NS Samarah AA : Prevalence of work-related musculoskeletal disorders and ergonomic practice among dentists in Jeddah, Saudi Arabia. *Clin. Cosmet. Investig. Dent.* 2019;Volume 11:171–179. 10.2147/CCIDE.S204433 31308760 PMC6615716

[46] Harris ML Sentner SM Doucette HJ : Musculoskeletal disorders among dental hygienists in Canada. *Can. J. Dent. Hyg.* 2020;54(2):61–67. 33240365 PMC7668274

[47] Al Muraikhi SSHS Rahimi KEME Shetty AC : Prevalence of clinical signs of carpel tunnel syndrome among dentist in Qatar. *Eur. J. Mol. Clin. Med.* 2020;7(7):450–456.

[48] Macdonald G Robertson MM Erickson JA : Carpal tunnel syndrome among California dental hygienists. *Dent. Hyg (Chic).* 1988;62(7):322–327. 3215371

[49] Osborn JB Newell KJ Rudney JD : Carpal tunnel syndrome among Minnesota dental hygienists. *J. Dent. Hyg.* 1990;64(2):79–85. 2370585

[50] Conrad JC Conrad KJ Osborn JS : Median nerve dysfunction evaluated during dental hygiene education and practice (1986-1989). *J. Dent. Hyg.* 1991;65(6):283–288. 1819629

[51] Conrad JC Conrad KJ Osborn JB : A short-term epidemiological study of median nerve dysfunction in practicing dental hygienists. *J. Dent. Hyg.* 1992;66(2):76–80. 1624996

[52] Feng B Chen K Zhu X : Prevalence and risk factors of self-reported wrist and hand symptoms and clinically confirmed carpal tunnel syndrome among office workers in China: a cross-sectional study. *BMC Public Health.* 2021;21(1):57. 10.1186/s12889-020-10137-1 33407293 PMC7789363

[53] Hagberg M Morgenstern H Kelsh M : Impact of occupations and job tasks on the prevalence of carpal tunnel syndrome. *Scand. J. Work Environ. Health.* 1992;18(6):337–345. 10.5271/sjweh.1564 1485158

[54] Atroshi I : Prevalence of Carpal Tunnel Syndrome in a General Population. *JAMA.* 1999;282(2):153. 10.1001/jama.282.2.153 10411196

[55] Andersen JH Thomsen JF Overgaard E : Computer use and carpal tunnel syndrome: a 1-year follow-up study. *JAMA.* 2003;289(22):2963–2969. 10.1001/JAMA.289.22.2963 12799404

[56] Lee IH Kim YK Kang DM : Distribution of age, gender, and occupation among individuals with carpal tunnel syndrome based on the National Health Insurance data and National Employment Insurance data. *Ann. Occup. Environ. Med.* 2019;31(1):e31. 10.35371/aoem.2019.31.e31 31737286 PMC6850790

