# Supplementary material for: Worldwide prevalence of carpal tunnel syndrome among dental health care personnel - A systematic review and meta-analysis
Source: F1000Res. 2023 Jul 20;12:251. Originally published 2023 Mar 8. [Version 2] doi: 10.12688/f1000research.131659.2 (PMC10372462; doi:10.12688/f1000research.131659.2)
Supplement: Peer review history

# Peer review: Reviewer response for version 2

**DOI:** 10.5256/f1000research.152898.r188751

**Published:** 2023-07-26

**Authors:** Dileep Nag Vinnakota, Rekhalakshmi Kamatham

**Affiliations:** 1 Department of Prosthodontics, Narayana Dental College, Nellore, Andhra Pradesh, India; 2 Department of Paedodontics and Preventive Dentistry, Narayana Dental College, Nellore, Andhra Pradesh, India

The revisions made by the authors satisfactorily address our comments, and based on their efforts, I recommend approving the article for indexing.

Are the rationale for, and objectives of, the Systematic Review clearly stated?

Yes

Is the statistical analysis and its interpretation appropriate?

Yes

Are sufficient details of the methods and analysis provided to allow replication by others?

Partly

Are the conclusions drawn adequately supported by the results presented in the review?

Partly

Reviewer Expertise:

Implantology, Full mouth rehabilitation

We confirm that we have read this submission and believe that we have an appropriate level of expertise to confirm that it is of an acceptable scientific standard.

# Peer review: Reviewer response for version 2

**DOI:** 10.5256/f1000research.152898.r188750

**Published:** 2023-07-20

**Authors:** Y. Ravi shankar Reddy

**Affiliations:** 1 Department of Medical Rehabilitation Sciences, College of Applied Medical Sciences, King Khalid University, Abha, Aseer Province, Saudi Arabia

I believe the authors have effectively responded to the reviewers' comments in the revised manuscript, so I have no additional remarks to add.

Are the rationale for, and objectives of, the Systematic Review clearly stated?

Yes

Is the statistical analysis and its interpretation appropriate?

Partly

Are sufficient details of the methods and analysis provided to allow replication by others?

Partly

Are the conclusions drawn adequately supported by the results presented in the review?

Yes

Reviewer Expertise:

Musculosketal Rehabilitation

I confirm that I have read this submission and believe that I have an appropriate level of expertise to confirm that it is of an acceptable scientific standard.

# Peer review: Reviewer response for version 1

**DOI:** 10.5256/f1000research.144521.r178238

**Published:** 2023-06-19

**Authors:** Y. Ravi shankar Reddy

**Affiliations:** 1 Department of Medical Rehabilitation Sciences, College of Applied Medical Sciences, King Khalid University, Abha, Aseer Province, Saudi Arabia

Title: Meta-analysis of the prevalence of Carpal Tunnel Syndrome (CTS) among dental health care personnel. 

1. Provide a clear rationale for conducting the study and highlight the gap in the existing literature that the study aims to address.
2. Clearly define the inclusion and exclusion criteria for the selection of studies to ensure transparency and reproducibility.
3. Discuss the methods used for data extraction and risk of bias assessment in more detail, including the specific items evaluated in the risk of bias assessment tool.
4. Address the high level of heterogeneity among the included studies and discuss potential reasons for this heterogeneity. Explore possible sources of variation, such as differences in study design, participant characteristics, diagnostic methods, or geographic location.
5. Provide a more comprehensive discussion of the limitations of the study, including the potential impact of publication bias, language restriction, and the quality of the included studies.
6. Discuss the implications of the findings in more depth, including the potential impact of CTS on the quality of life, functional disability, and productivity of dental healthcare personnel.
7. Consider the potential confounding factors or interactions that could influence the relationship between CTS and dental healthcare personnel, such as the duration of practice, specific dental procedures performed, or the use of ergonomic interventions.
8. Provide recommendations for future research based on the identified limitations and gaps in knowledge. Discuss the need for studies with larger sample sizes, more rigorous study designs, and standardized diagnostic criteria.
9. Discuss the practical implications of the findings and potential interventions that can be implemented to reduce the risk of CTS among dental healthcare personnel. Consider the development of guidelines for ergonomic practices, workload management, and education on early detection and prevention of CTS.
10. Ensure that the conclusion aligns with the findings of the study and emphasizes the key takeaways for researchers, clinicians, and policymakers.
11. Consider the broader implications of the study findings, such as the potential impact on healthcare policy, occupational health and safety regulations, and professional training programs for dental healthcare personnel.
12. Finally, acknowledge any limitations of the study not previously discussed and provide suggestions for further research in the field of CTS among dental healthcare personnel.

Are the rationale for, and objectives of, the Systematic Review clearly stated?

Yes

Is the statistical analysis and its interpretation appropriate?

Partly

Are sufficient details of the methods and analysis provided to allow replication by others?

Partly

Are the conclusions drawn adequately supported by the results presented in the review?

Yes

Reviewer Expertise:

Musculosketal Rehabilitation

I confirm that I have read this submission and believe that I have an appropriate level of expertise to confirm that it is of an acceptable scientific standard, however I have significant reservations, as outlined above.

# Response

**Published:** 2023-07-10

**Authors:** Kalyana Pentapati

**Affiliations:** Manipal Academy of Higher Education, India

We thank your efforts in reviewing the manuscript. Below are the responses for your comments. 

**Query:** Provide a clear rationale for conducting the study and highlight the gap in the existing literature that the study aims to address.

**Response:** We added the following information: Data pertaining to the pooled prevalence of CTS and associated risk factors is lacking among dental healthcare personnel.

**Query: **Clearly define the inclusion and exclusion criteria for the selection of studies to ensure transparency and reproducibility.

**Response:** we have revised as follows: Studies written in English that reported the prevalence of CTS using self-reported or clinical tests or NCS or where the prevalence could be determined were included. Studies reported as letters, commentaries, and short communications were excluded.

**Query: **Discuss the methods used for data extraction and risk of bias assessment in more detail, including the specific items evaluated in the risk of bias assessment tool.

**Response:** Revised the information as below:

 Two review authors independently performed the data extraction. Information that was collected was authors, year of publication, country, type of dental personnel (Dentist, dental auxiliary or Mixed population), age and sex distribution, age, sex and clinical experience distribution with respect to the distribution of CTS, sample size, number of participants with CTS, method of diagnosis used (self-reported, clinical examination, or NCS), the sex distribution of CTS and risk of bias.

 The first four questions (representative target population and sampling frame, random selection and non-response bias) assessed the external validity and the later five questions (participant or proxy data collection, acceptable case definition, validity and reliability of the instrument, similar data collection for all participants and specifying the numerators and denominators) assessed the internal validity of the study. All the questions were rated as low or high risk.

**Query: **Address the high level of heterogeneity among the included studies and discuss potential reasons for this heterogeneity. Explore possible sources of variation, such as differences in study design, participant characteristics, diagnostic methods, or geographic location.

**Response: **We attempted to explore the reasons for heterogeneity using sub-group analysis. The nature of the study design was same for all the studies. With respect to participant characteristics, we analysed based on the type of dental health care personnel which also showed high heterogeneity. Similar, was the case with geographic location. With respect to type of diagnosis, clinical examination showed lower levels of heterogeneity (<50%) than other modalities of diagnosis. However, the number of studies that used clinical examination were less (n=5).

**Query: **Provide a more comprehensive discussion of the limitations of the study, including the potential impact of publication bias, language restriction, and the quality of the included studies.

**Response: **Added the following information: Publication bias was due to less precise studies with high prevalence estimates which could have distorted the overall estimates. Language bias due to inclusion of studies that were reported in English could have over or under-estimated the overall pooled estimates. Although, most of the studies had low risk of bias, the lack of acceptable diagnostic standard could have caused considerable heterogeneity.

**Query:** Discuss the implications of the findings in more depth, including the potential impact of CTS on the quality of life, functional disability, and productivity of dental healthcare personnel.

**Response:** Added the following information: Implications of CTS can be at an individual, family or workplace level. Symptoms like pain, numbness, tingling and weakness in the hand and fingers can lead to significant functional disability to the dental personnel while performing the dental procedures. There by substantially affecting the individuals’ quality of life in performing daily activities and decreased productivity at workplace leading to loss of work, work place absenteeism along with financial losses. Indirectly, employers can face decreased productivity and loss of time in hiring new professionals. This can affect the overall productivity. Moreover, CTS can have a profound impact on the outside of the work, affecting simple tasks like writing, typing and grasping objects which may become challenging and be a limitation in their professional and personal activities alike. 

**Query: **Consider the potential confounding factors or interactions that could influence the relationship between CTS and dental healthcare personnel, such as the duration of practice, specific dental procedures performed, or the use of ergonomic interventions.

**Response: **We agree with reviewers about the role of potential confounders. We have included the data specific to age and clinical experience with respect to CTS. However, data on other factors like type of dental procedures and use of ergonomic interventions have not been reported in relation to the CTS prevalence among the included studies. It is crucial for further research to account for these confounding factors and assess their relationship between CTS and dental healthcare personnel.

**Query: **Provide recommendations for future research based on the identified limitations and gaps in knowledge. Discuss the need for studies with larger sample sizes, more rigorous study designs, and standardized diagnostic criteria.

**Response:** Added information as below:

 Further large high-quality studies using clinical examination for the identification of CTS among a representative sample of dental health care personnel using STROBE guidelines are required for calculating robust prevalence estimates. A larger sample would allow for a more representative distribution of demographic and professional characteristics, enabling researchers to explore potential subgroups that may be more susceptible to CTS. Also, a larger sample size would provide more statistical power to detect significant associations and to examine the effects of potential confounding factors. Case control studies are required to understand the role of dental profession as risk factor for the development of CTS. Studies can use self-reported questionnaires to screen potential participants following which a clinical examination using various tests need to be adopted to diagnose CTS.  Furthermore, adapting standardized, validated diagnostic criteria for CTS across studies would facilitate more apt comparisons of different studies and enhance the reliability of the study findings.

**Query:** Discuss the practical implications of the findings and potential interventions that can be implemented to reduce the risk of CTS among dental healthcare personnel. Consider the development of guidelines for ergonomic practices, workload management, and education on early detection and prevention of CTS.

 During training years, emphasis should be on the potential role of dental profession in the development of CTS and other musculoskeletal disorders. There is need for the development and implementation of preventive strategies for early detection and prevention of CTS. Comprehensive preventive strategies like workplace postural requirements and adoption of ergonomic postures, use of ergonomically designed instruments and equipment to reduce strain on the hand and wrist, the importance of intermittent breaks between patients, an alternation between the activities, keeping wrists in a neutral position, strengthening and stretching daily which aid in alleviating the muscular tension and promote blood circulation, minimize repetitive movements and management of patient flow can be incorporated into the curriculum during training years to prevent or minimise the onset of musculoskeletal disorders. Regular monitoring and evaluation of musculoskeletal disorders need to be mandated for high-risk individuals. Workplace-associated CTS must be identified earlier and care should be exercised on the prevention and progression of the development of CTS. Active referral should be initiated by the employer who is at risk of development of CTS. It is important for the dental health care professional to be aware of the symptoms of CTS. Early diagnosis will help in initiating early interventions like medication, splinting, and changes in daily activities and can be relieved without surgical interventions. Therefore, a multi-pronged approach with ergonomic guidelines, workload management strategies, and health education and prevention can significantly reduce the risk of CTS among dental personnel and enhance their occupational well-being.

**Query: **Ensure that the conclusion aligns with the findings of the study and emphasizes the key takeaways for researchers, clinicians, and policymakers.

**Response:** Future studies should explore the relationship between various potential risk factors and CTS. There is a need to develop and incorporate guidelines for the prevention of work-related musculoskeletal disorders into the training curriculum. Continuing dental education programs for the prevention of musculoskeletal disorders need to be conducted for the benefit of dental health care personnel.

**Query:** Consider the broader implications of the study findings, such as the potential impact on healthcare policy, occupational health and safety regulations, and professional training programs for dental healthcare personnel.

**Response: **Findings of this review highlight the potential impact of CTS among dental healthcare personnel. Policymakers need to ensure the development and implementation of the guidelines for the prevention of work-related musculoskeletal disorders and the incorporation of the same into the curriculum. Occupational Safety and Health Administration guidelines on the prevention of work-related musculoskeletal disorders have elements like management support, involvement of staff, training, identification of problems, early reporting, solutions to control hazards and evaluation. It should also emphasize the importance of the number of patients seen and attended, periodic breaks, task rotation, workflow and workload in the prevention of musculoskeletal disorders.

**Query: **Finally, acknowledge any limitations of the study not previously discussed and provide suggestions for further research in the field of CTS among dental healthcare personnel.

**Response: **The information below was added:

 Further large high-quality studies using clinical examination for the identification of CTS among a representative sample of dental health care personnel using STROBE guidelines are required for calculating robust prevalence estimates. A larger sample would allow for a more representative distribution of demographic and professional characteristics, enabling researchers to explore potential subgroups that may be more susceptible to CTS. Also, a larger sample size would provide more statistical power to detect significant associations and to examine the effects of potential confounding factors. Case control studies are required to understand the role of the dental profession as a risk factor for the development of CTS. Studies can use self-reported questionnaires to screen potential participants following which a clinical examination using various tests need to be adopted to diagnose CTS.  Furthermore, adapting standardized, validated diagnostic criteria for CTS across studies would facilitate more apt comparisons of different studies and enhance the reliability of the study findings.

 High heterogeneity among the included studies, inclusion of only studies that were reported in English, publication bias, lack of age specific estimates, and variations in the assessment of CTS are some of the limitations. Publication bias was due to less precise studies with high prevalence estimates which could have distorted the overall estimates. Language bias due to the inclusion of studies that were reported in English could have over or under-estimated the overall pooled estimates. Although, most of the studies had low risk of bias, the lack of acceptable diagnostic standards could have caused considerable heterogeneity.

# Peer review: Reviewer response for version 1

**DOI:** 10.5256/f1000research.144521.r165926

**Published:** 2023-03-24

**Authors:** Dileep Nag Vinnakota, Rekhalakshmi Kamatham

**Affiliations:** 1 Department of Prosthodontics, Narayana Dental College, Nellore, Andhra Pradesh, India; 2 Department of Paedodontics and Preventive Dentistry, Narayana Dental College, Nellore, Andhra Pradesh, India

I have gone through the meta-analysis sent for peer review. The topic seems interesting, but there are lot of flaws which are of concern. I have mentioned the possibilities of changing the manuscript section wise.

 Title: The authors have mentioned the title as “Meta-analysis of the prevalence of carpal tunnel syndrome among dental health care personnel”. I feel the title can be improved as “Prevalence of carpal tunnel syndrome among dental health care personnel worldwide - A systematic review and meta-analysis”

 Introduction: The need for the study has been focused by the authors properly. However, can elaborate on the clinical diagnosis of the condition (including the sensitivity and specificity of the tests), and a note on the differential diagnosis. A systematic review is also published on the diagnosis of this condition. This part is important as it has a link with the way the authors can mold the quality of the present systematic review.

 Methodology: Many sections are poorly written.

- The authors can mention the keywords separately as MeSH words and alternate words employed for the search.
- The inclusion and exclusion criteria are not proper. The authors can consider only those studies that have employed nerve conduction tests, instead of considering those with self-report. There are definite chances of bias, if the self-reported measures are considered for diagnosis. Also many factors like number of years practised in dentistry, number of working hours, the knowledge of ergonomics, and age of the dental personnel all play a key role on prevalence. The authors did not take any step to cut down the effect of confounding factors in their criteria for inclusion.
- Under extraction of data, in text, the authors have mentioned that ‘information regarding country of study’, but in the table the authors have considered only continent.
- Under risk of bias assessment, the authors have mentioned that risk was evaluated using a nine item questionnaire. The authors have to elaborate the items in a tabular form along with the total scores obtained by individual studies. Need to mention the reason for not considering the STROBE guidelines for evaluation? I think they can add this part also.
- Under statistical analysis, the authors did not mention the test employed for the publication bias. Is it Begg and Egger test?

Results:

- It is clearly evident from Table 1 that the prevalence is more in studies that have diagnosed the condition using self-reported measures. The type of dental health care personnel also cannot be combined; the work done by dentists is different from dental assistants. The mean age as mentioned in the review is in the range of 21-50 years, which is too long. All these have an influence on the overall estimate mentioned.
- Clarity of the forest plot showing gender differences is not clear. The authors need to mention the sides as male and female, so that the readers can easily depict the side which is favoured.
- Under publication bias, the authors did not explain about the symmetry of the funnel plot. What is inverse standard error? Also, the reason for selecting the inverse standard error needs mentioning. The scatter plot needs explanation regarding the precision of individual studies.
- Under sensitivity analysis, the authors need to mention the outliers and the explanation of difference in the overall estimate after removal of studies with high prevalence like Prasad et al (2017).

Discussion: Discussion is not enough. Authors can discuss the importance of case control studies to know whether dentists are really more prone for this condition compared to normal population. Authors can discuss about the ergonomics to prevent these conditions. Can discuss about differential diagnosis of this condition and discuss about other neurologic, musculoskeletal and vascular conditions that have similar symptoms as carpal tunnel syndrome. Also, management of this condition (non-surgical and exercises) needs mentioning.

Are the rationale for, and objectives of, the Systematic Review clearly stated?

Yes

Is the statistical analysis and its interpretation appropriate?

Yes

Are sufficient details of the methods and analysis provided to allow replication by others?

Partly

Are the conclusions drawn adequately supported by the results presented in the review?

Partly

Reviewer Expertise:

Implantology, Full mouth rehabilitation

We confirm that we have read this submission and believe that we have an appropriate level of expertise to confirm that it is of an acceptable scientific standard, however we have significant reservations, as outlined above.

## References

[1] : Clinical diagnosis of carpal tunnel syndrome: a systematic review. *J Hand Ther* .2004;17(2) : 10.1197/j.jht.2004.02.015 309-19 10.1197/j.jht.2004.02.015 15162113

# Response

**Published:** 2023-07-10

**Authors:** Kalyana Pentapati

**Affiliations:** Manipal Academy of Higher Education, India

Thank you for the effort and time take in reviewing this manuscript. Below are our responses to your comments. 

 Reviewer 1:

 I have gone through the meta-analysis sent for peer review. The topic seems interesting, but there are lot of flaws which are of concern. I have mentioned the possibilities of changing the manuscript section wise.

 Title: The authors have mentioned the title as “Meta-analysis of the prevalence of carpal tunnel syndrome among dental health care personnel”. I feel the title can be improved as “Prevalence of carpal tunnel syndrome among dental health care personnel worldwide - A systematic review and meta-analysis”

**Response: **We will edit the same in our manuscript “Worldwide prevalence of carpal tunnel syndrome among dental health care personnel - A systematic review and meta-analysis”

 Introduction: The need for the study has been focused by the authors properly. However, can elaborate on the clinical diagnosis of the condition (including the sensitivity and specificity of the tests), and a note on the differential diagnosis. A systematic review is also published on the diagnosis of this condition. This part is important as it has a link with the way the authors can mold the quality of the present systematic review.

**Response: **Thank you for your valuable insight. We had gone through MacDermid and Wessel to understand the value-added points. They concluded that the primary limitation of establishing a gold standard diagnosis for CTS. This difficulty, combined with methodologic flaws, made interpretation difficult. Also, there was no guarantee that these choices increased the validity of our results. It was also stated that we should not consider the findings of this review to be conclusive concerning the value of clinical tests in the diagnosis of CTS. Furthermore, a systematic review requires that issues be addressed using specific and documented methods. Therefore, discussions on why certain tests may be better in specific situations or relevant in different stages of CTS are beyond the scope of this review.

 Our review focussed on the burden of disease (CTS) among dental health professionals which was by pooling the estimates that have been reported from the literature. Various methods are available in the literature for the diagnosis of CTS which are broadly categorized as self-reported, clinical, and nerve conduction studies. All these tests have their own advantages and limitations that are discussed by the authors of these primary studies.

 Methodology: Many sections are poorly written.

**Query: **The authors can mention the keywords separately as MeSH words and alternate words employed for the search.

**Response: **MeSH words are only specific for Pubmed. We have used All fields option for all the databases and have not restricted our search to title, abstract and keywords search. Based on our previous review experience on musculoskeletal disorders, we noticed that few studies that reported prevalence estimates of musculoskeletal disorders have reported carpal tunnel syndrome. If we restrict our search to title, abstract and keywords, these studies would be missed during the first stage of screening.  We will reframe the sentence as search terms instead of keywords in the manuscript.

**Query: **The inclusion and exclusion criteria are not proper. The authors can consider only those studies that have employed nerve conduction tests, instead of considering those with self-report. There are definite chances of bias, if the self-reported measures are considered for diagnosis. Also many factors like number of years practiced in dentistry, number of working hours, the knowledge of ergonomics, and age of the dental personnel all play a key role on prevalence. The authors did not take any step to cut down the effect of confounding factors in their criteria for inclusion.

**Response: **There is no consensus or gold standard for the diagnosis of carpal tunnel syndrome. Nerve conduction studies are a useful tool and can be used as complementary methods with clinical examination in the assessment of CTS. Use of such methods in epidemiological surveys is quite challenging as it is costly, requires trained personnel to perform and interpret and cannot be used alone for the diagnosis of CTS. Hence, there were many attempts to develop self-reported measures and clinical examination for the diagnosis of CTS. Self-reported measures like boston carpal tunnel questionnaire has been used widely and was shown to have acceptable validity and reliability in many previous studies. We agree that there were will be bias, hence we reported the estimates of CTS as per the diagnostic method used in the primary studies.

 We have revised the sentence: Studies written in English that reported the prevalence of CTS using self-reported or clinical tests or NCS or where the prevalence could be determined were included. Studies reported as letters, commentaries, and short communications were excluded.

 We agree with reviewers that there are many factors (years practiced in dentistry, number of working hours, the knowledge of ergonomics, and age of the dental personnel) on the prevalence estimates. The main aim of this review was to quantify the prevalence estimates obtained from the published literature. To look at these factors that may have the effect of confounding, it is imperative that published literature reports that prevalence estimates as per age groups, work experience, level of ergonomics, etc. We have reviewed the included studies and extracted data on age, clinical experience, and gender distribution as per the CTS distribution.

**Response: **We will include the information below in the manuscript.

 Based on the data from the included studies, age estimates with respect to CTS and No CTS was reported by only six studies. Studies that reported age as categorical data could not be analysed as there was no similarity in the categories used (Table 2). Three studies reported age as continuous variable. Meta-analysis showed that there was no significant difference in the age between CTS and No CTS groups (SMD: 0.1; 95%CI: -0.17 – 0.38) (figure 3).

Image 1; Image 2

 A total of seven studies reported clinical experience which was either continuous (n=3) or categorical (n=4). Meta-analysis showed that there was no significant difference in the mean clinical experience between the groups (SMD: -0.03; 95%CI: -0.31 – 0.24) (Figure 5). Categorical data on clinical experience was categorized as < 10 years and > 10 years for analysis. Meta-analysis showed no significant difference in the pooled estimates between different levels of clinical experience (OR: 0.76; 95%CI: 0.39-1.47) (Figure 6).

Image 3; Image 4

**Query: **Under extraction of data, in text, the authors have mentioned that ‘information regarding country of study’, but in the table the authors have considered only continent.

**Response:** Yes. We have collected the information on country. However, country wise estimates were not feasible. Hence, we pooled the estimates as per the continent.

**Query: **Under risk of bias assessment, the authors have mentioned that risk was evaluated using a nine-item questionnaire. The authors have to elaborate the items in a tabular form along with the total scores obtained by individual studies. Need to mention the reason for not considering the STROBE guidelines for evaluation? I think they can add this part also.

**Response: **Thank you for your comments. We will incorporate the item-wise scores for individual studies. The questionnaire used for quality assessment was validated and primarily developed for the assessment of risk of bias for systematic reviews on prevalence studies. STROBE guidelines are primarily intended as a checklist for reporting cross-sectional studies. The valid overall risk of bias scores cannot be computed so that studies can be rated as high or low risk of bias from STROBE guidelines. Hence, it was not used in this study.

Image 5

**Query: **Under statistical analysis, the authors did not mention the test employed for the publication bias. Is it Begg and Egger test?

**Response:** We have used Fail-Safe N analysis using the Rosenthal approach. We will add this information also in the manuscript.

 Statistical analysis: Publication bias was assessed using a funnel plot and Fail-Safe N analysis using the Rosenthal approach and Egger regression test.

 Publication bias: Egger Regression Test for Funnel Plot Asymmetry showed asymmetry (Z=2.187; P=0.029).

**Query:** It is clearly evident from Table 1 that the prevalence is more in studies that have diagnosed the condition using self-reported measures. The type of dental health care personnel also cannot be combined; the work done by dentists is different from dental assistants. The mean age as mentioned in the review is in the range of 21-50 years, which is too long. All these have an influence on the overall estimate mentioned.

**Response: **Yes, we agree with the reviewer that self-reported measures have yielded higher estimates and we have reported the estimates as per the diagnostic criteria used in the studies. We also agree with the fact that type of dental health care personnel cannot be combined as all have different range of work but all these personnel do activities that requires repetitive movements, firm grasp, and fine tactile movements with prolonged static postures. Many studies which were included in this review have included all types of dental health personnel. Studies that reported prevalence separately were captured separately. In our review, we have mentioned the estimates as per the type of healthcare personnel. We understand that all these factors along with age and clinical experience have potential influence. This review is focussed on the burden of CTS among these dental health care personnel.

**Query: **Clarity of the forest plot showing gender differences is not clear. The authors need to mention the sides as male and female so that the readers can easily depict the side which is favoured.

**Response:** We will edit the figure in the manuscript as per your recommendation.

Image 6

**Query: **Under publication bias, the authors did not explain about the symmetry of the funnel plot. What is inverse standard error? Also, the reason for selecting the inverse standard error needs mentioning. The scatter plot needs an explanation regarding the precision of individual studies.

**Response:** We will add the following details to the manuscript.

 Inverse standard error in the y-axis depicts the precision of the studies. It helps in identifying the studies with lower precision which will be distributed at the bottom. Inverse standard error was selected as there was no inversion required when compared to plots that use standard error in the y-axis where studies with large sample sizes and lower standard error are placed at the top of the graph. The plot showed asymmetry where in large studies showed higher precision and lower prevalence estimates whereas smaller studies had lower precision and higher prevalence estimates.

**Query: **Under sensitivity analysis, the authors need to mention the outliers and the explanation of difference in the overall estimate after the removal of studies with high prevalence like Prasad et al (2017).

**Response: **We performed a sensitivity analysis using Leave -one out method. The prevalence estimate marginally decreased to 13% after the removal of Prasad et al. We will add this information in the manuscript.

**Query: **Discussion is not enough. Authors can discuss the importance of case-control studies to know whether dentists are really more prone to this condition compared to the normal population. Authors can discuss the ergonomics to prevent these conditions. Can discuss about differential diagnosis of this condition and discuss about other neurologic, musculoskeletal and vascular conditions that have similar symptoms as carpal tunnel syndrome. Also, the management of this condition (non-surgical and exercises) needs mentioning.

**Response: **The below information will be added in the manuscript.

 Further case-control studies are required to understand the role of the dentistry profession as a risk factor for the development of CTS are needed.

 During training years, emphasis should be on the potential role of the dental profession in the development of CTS and other musculoskeletal disorders. There is a need for the development and implementation of preventive strategies for early detection and prevention of CTS. Comprehensive preventive strategies like workplace postural requirements and adoption of ergonomic postures, use of ergonomically designed instruments and equipment to reduce strain on the hand and wrist, the importance of intermittent breaks between patients, an alternation between the activities, keeping wrists in a neutral position, strengthening and stretching daily which aid in alleviating the muscular tension and promote blood circulation, minimize repetitive movements and management of patient flow can be incorporated into the curriculum during training years to prevent or minimize the onset of musculoskeletal disorders. Regular monitoring and evaluation of musculoskeletal disorders need to be mandated for high-risk individuals. Workplace-associated CTS must be identified earlier, and care should be exercised on the prevention and progression of the development of CTS. Active referral should be initiated by the employer who is at risk of development of CTS. It is important for the dental health care professional to be aware of the symptoms of CTS.

 Many conditions like systemic neurologic disorders (motor neuron disease, multiple sclerosis, and hereditary neuropathy), cervical spine disorders (cervical spondylotic myelopathy, cervical radiculopathy, and syringomyelia), tumors (Pancoast tumors, benign peripheral nerve tumors, malignant peripheral nerve sheath tumors, intraneural ganglia), inflammatory and autoimmune disorders.  

 The management of CTS includes conservative methods like wrist splinting in the neutral position at night, analgesics, corticosteroid injections, and nerve and tendon gliding exercises with varying degrees of results. Also, the carpal tunnel can be surgically decompressed to relieve the symptoms when there is a lack of response to the above. Early diagnosis will help in initiating early interventions like medication, splinting, and changes in daily activities and can be relieved without surgical interventions. Therefore, a multi-pronged approach with ergonomic guidelines, workload management strategies, and health education and prevention can significantly reduce the risk of CTS among dental personnel and enhance their occupational well-being.
